# Electrosurgical-laceration and stabilization of two PASCAL devices using artificial intelligence-based procedural planning: a case report

**DOI:** 10.1093/ehjcr/ytaf202

**Published:** 2025-04-25

**Authors:** Stephan Nienaber, Jonathan Curio, Elmar W Kuhn, Hendrik Wienemann, Matti Adam

**Affiliations:** Department of Cardiology, Heart Center Cologne, University of Cologne, Faculty of Medicine and University Hospital, Kerpener Straße 62, 50937 Cologne, Germany; Department of Cardiology, Heart Center Cologne, University of Cologne, Faculty of Medicine and University Hospital, Kerpener Straße 62, 50937 Cologne, Germany; Department of Heart Surgery, Heart Center Cologne, University of Cologne, Faculty of Medicine and University Hospital, Kerpener Straße 62, 50937 Cologne, Germany; Department of Cardiology, Heart Center Cologne, University of Cologne, Faculty of Medicine and University Hospital, Kerpener Straße 62, 50937 Cologne, Germany; Department of Cardiology, Heart Center Cologne, University of Cologne, Faculty of Medicine and University Hospital, Kerpener Straße 62, 50937 Cologne, Germany

**Keywords:** Mitral regurgitation, Recurrent, ELASTA-Clip, Mitral valve lifetime management, Case report

## Abstract

**Background:**

Mitral transcatheter edge-to-edge repair (M-TEER) has been established as a treatment for severe mitral regurgitation (MR). However, recurrent MR may occur after successful primary M-TEER. Repeat M-TEER procedures are challenging or even anatomically unfeasible. Therefore, novel techniques like electrosurgical laceration from the anterior leaflet and stabilization (ELASTA) of M-TEER devices are important.

**Case summary:**

An 85-year-old male presented with recurrent cardiac decompensations and relevant dyspnoea [New York Heart Association (NYHA) functional Class III–IV] caused by severe MR and heart failure. Four years earlier, the patient underwent M-TEER with two PASCAL devices due to severe primary MR. Echocardiography revealed two significant regurgitation jets, representing severe recurrent MR. Repeated M-TEER was prohibitive due to an elevated mean transvalvular gradient (6.2 mmHg) and surgery was deemed unfeasible. A computed tomography (CT) was performed using software based on artificial intelligence (AI) for automatic analysis of the mitral valve anatomy. The heart team then decided to perform an ELASTA of the two PASCAL devices with subsequent transapical transcatheter mitral valve implantation (TMVI). The procedure was performed without complications and achieved optimal haemodynamic outcomes.

**Discussion:**

The frequency of M-TEER procedures will rise, leading to a corresponding increase of recurrent MR requiring treatment. However, additional edge-to-edge repair is often not feasible due to space limitations, elevated inflow gradients, or progressive calcification of valve leaflets. Emerging electrosurgical techniques, such as ELASTA, combined with subsequent TMVI, offer promising solutions for recurrent MR. Computed tomography imaging plays a critical role in procedural planning, and AI-driven programs have the potential to significantly improve and optimize these analyses.

**Learning points**

- Precise pre-procedural imaging-based planning in complex transcatheter mitral valve interventions is essential and may be facilitated by artificial intelligence (AI)-based software.
- Potential techniques, such as electrosurgical laceration and stabilization (ELASTA), allow for a lifetime management beyond a first intervention in patients with mitral valve disease who underwent mitral transcatheter edge-to-edge repair (M-TEER).

## Introduction

Mitral transcatheter edge-to-edge repair (M-TEER) has become an established part of the treatment armamentarium for patients with severe mitral regurgitation (MR) at increased surgical risk.^1^ Nevertheless, recurrent MR may occur after successful primary M-TEER. Repeat M-TEER procedures are technically challenging or even anatomically unfeasible. Electrosurgical laceration from the anterior leaflet and stabilization (ELASTA) of M-TEER devices has been introduced as novel technique to cut the mitral tissue bridge, enabling subsequent transcatheter mitral valve implantation (TMVI).^2^ To further refine and standardize these procedures, meticulous pre-procedural planning is essential, potentially streamlined by automatic artificial intelligence (AI)-based assessment.

## Summary figure

Electrosurgical laceration and stabilization (ELASTA) of two PASCAL devices with AI-based pre-procedural planning. AI, artificial intelligence; ELASTA, electrosurgical laceration and stabilization; MR, mitral regurgitation; TMVI, transcatheter mitral valve implantation.

**Figure:**
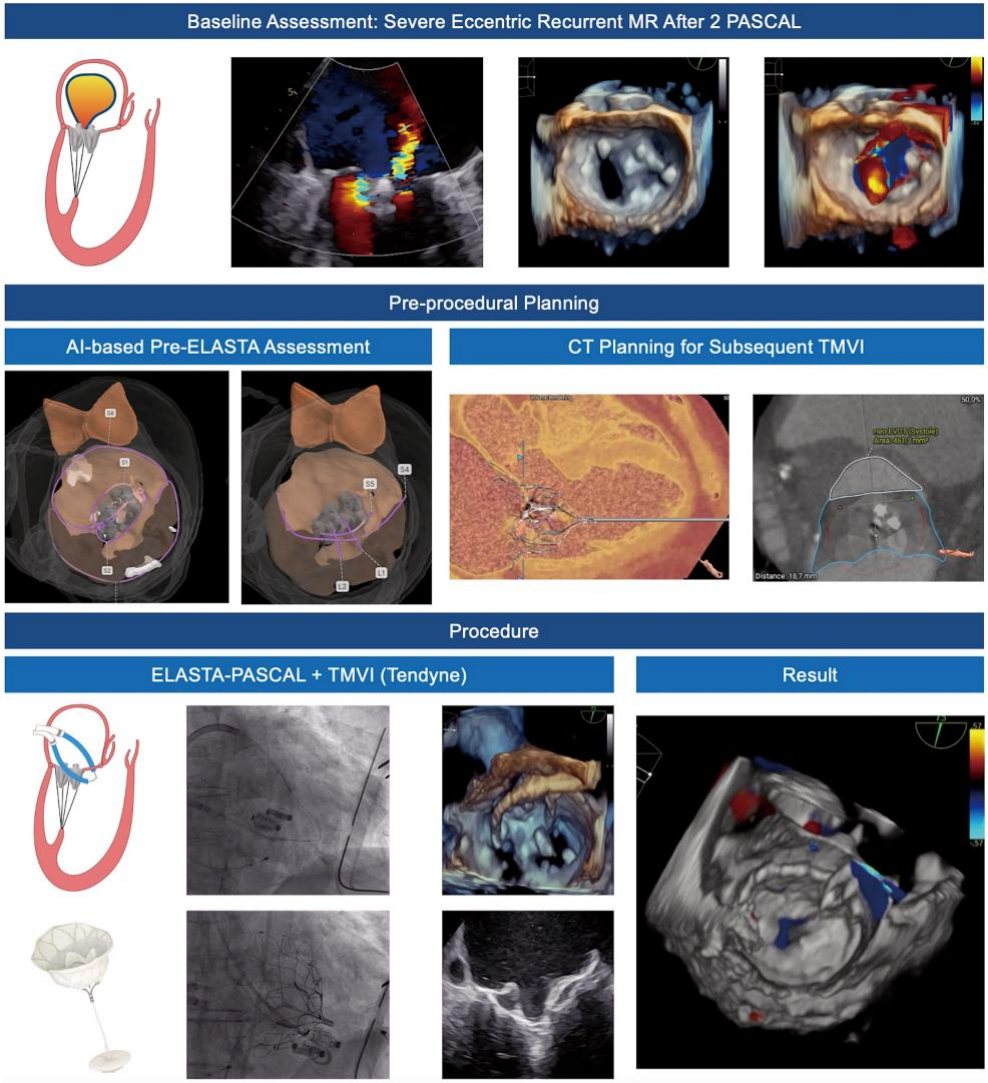


## Case presentation

An 85-year-old male of Caucasian ethnicity was referred to our centre following recurrent cardiac decompensation, suffering from relevant exhaustion and dyspnoea [New York Heart Association (NYHA) functional Class III–IV] caused by severe MR and heart failure with mildly reduced ejection fraction (41%). Echocardiography revealed severe MR as the only valvular pathology.

The patient had a history of single-vessel-coronary artery disease, heart failure, chronic renal insufficiency with a glomerular filtration rate of 49 mL/min, and arterial hypertension. Moreover, the patient suffered from chronic obstructive lung disease and was on oral anticoagulation therapy due to atrial fibrillation and a history of pulmonary embolism. Importantly, the patient underwent mitral valve transcatheter edge-to-edge repair (M-TEER) due to severe primary MR caused by a flail of the posterior mitral valve leaflet (PML) 4 years earlier. The implantation of two PASCAL (Edwards Lifesciences, Irvine, CA, USA) devices was required to achieve a satisfactory outcome with only mild residual MR.

**Figure 1:**
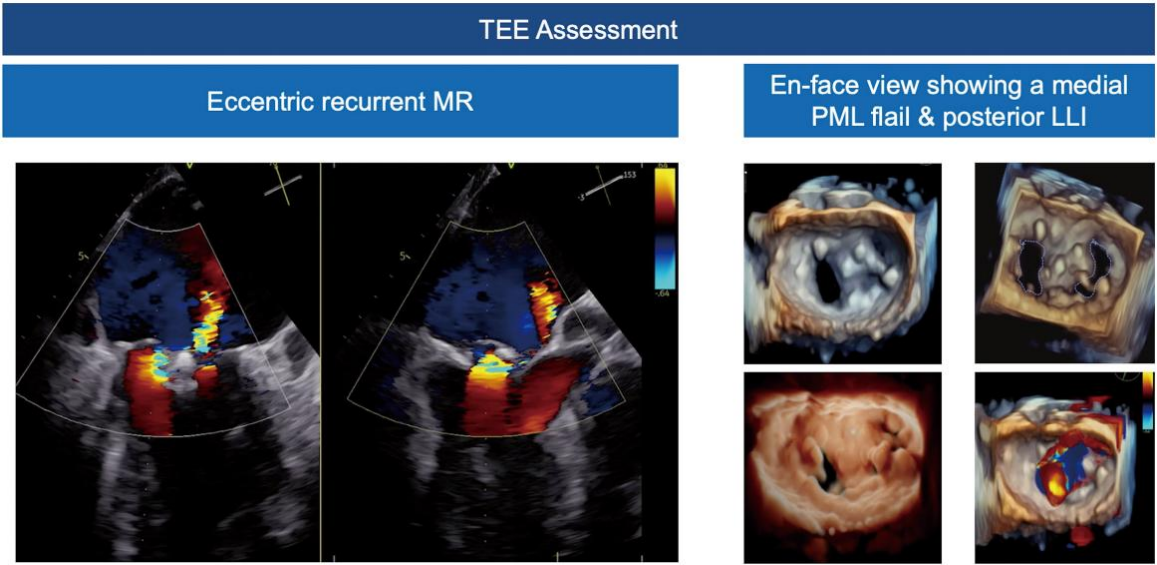
Baseline echocardiography showing recurrent mitral regurgitation after mitral transcatheter edge-to-edge repair with two PASCAL. LLI, loss of leaflet insertion; MR, mitral regurgitation; M-TEER, mitral transcatheter edge-to-edge repair; PML, posterior mitral leaflet; TEE, transesophageal echocardiography.

Physical examination revealed peripheral oedema and a holodiastolic murmur with a point of maximal intensity over the fifth intercostal space. Echocardiography showed a slightly dilated left ventricle (left ventricular end-diastolic diameter: 51 mm, left ventricular end-systolic diameter: 45 mm) with reduced ejection fraction (41%). Both previously implanted PASCAL devices were in place and a detachment was ruled out by transoesophageal echocardiography (TEE). However, TEE unveiled a flail of the PML medial to the two PASCAL devices (*Figure 1*). Another significant regurgitation jet was located lateral to the PASCAL devices, caused by a coaptation defect, collectively representing recurrent severe MR. Repeated M-TEER was prohibitive due to a heightened mean transvalvular gradient (6.2 mmHg) assessed using TEE.^3^ Considering pre-existing comorbidities and an elevated surgical risk [Society of Thoracic Surgeons (STS) score of 7.22%], the local heart team decided to perform an ELASTA of the two PASCAL devices with subsequent transapical TMVI using the tether-based Tendyne (Abbott Vascular, Abbott Park, IL, USA) prosthesis.

**Figure 2:**
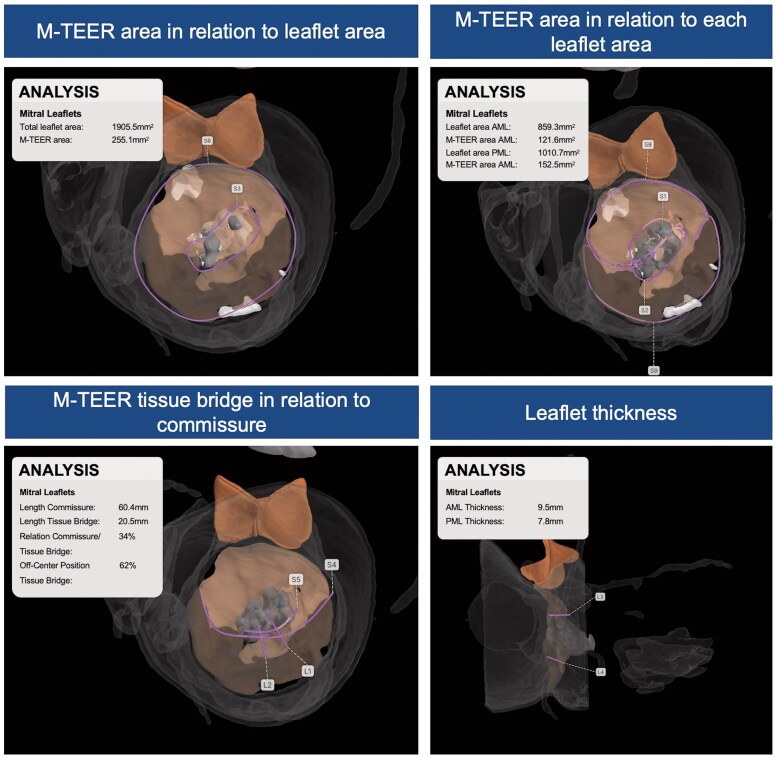
Artificial intelligence-based assessment of electrosurgical laceration and stabilization-specific parameters for precise procedural planning. AI, artificial intelligence; ELASTA, electrosurgical laceration and stabilization; M-TEER, mitral transcatheter edge-to-edge repair.

**Figure 3:**
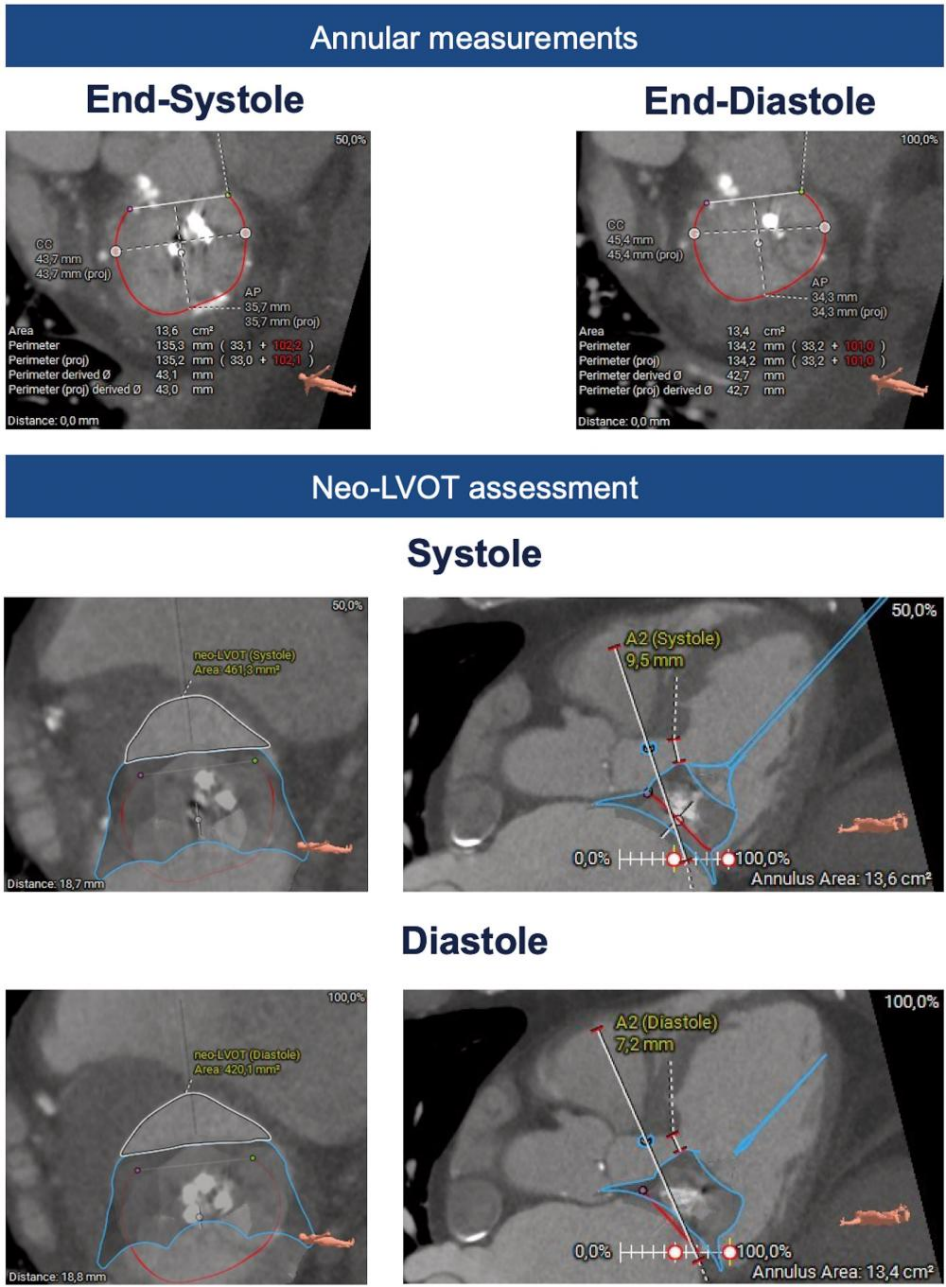
Pre-procedural planning of subsequent transcatheter mitral valve implantation after electrosurgical laceration and stabilization-PASCAL. ELASTA, Electrosurgical laceration and stabilization; LVOT, left ventricular outflow tract; TMVI, transcatheter mitral valve implantation.

A computed tomography (CT) was performed to run an automatic analysis of the mitral valve anatomy using AI-based software (LARLAB GmbH, Munich, Germany), to understand the potential challenges of the ELASTA procedure in detail (*Figures 2* and *3*, Supplementary material online, *Video S1*). Based on the AI-generated measurements, ELASTA with the two PASCAL devices present was considered feasible, due to a sufficient size of the anterior mitral valve leaflet (AML), adequate attachment of the devices to the PML, and the volume of the M-TEER package likely fitting into a posterior pocket of the ventricle.

**Figure 4:**
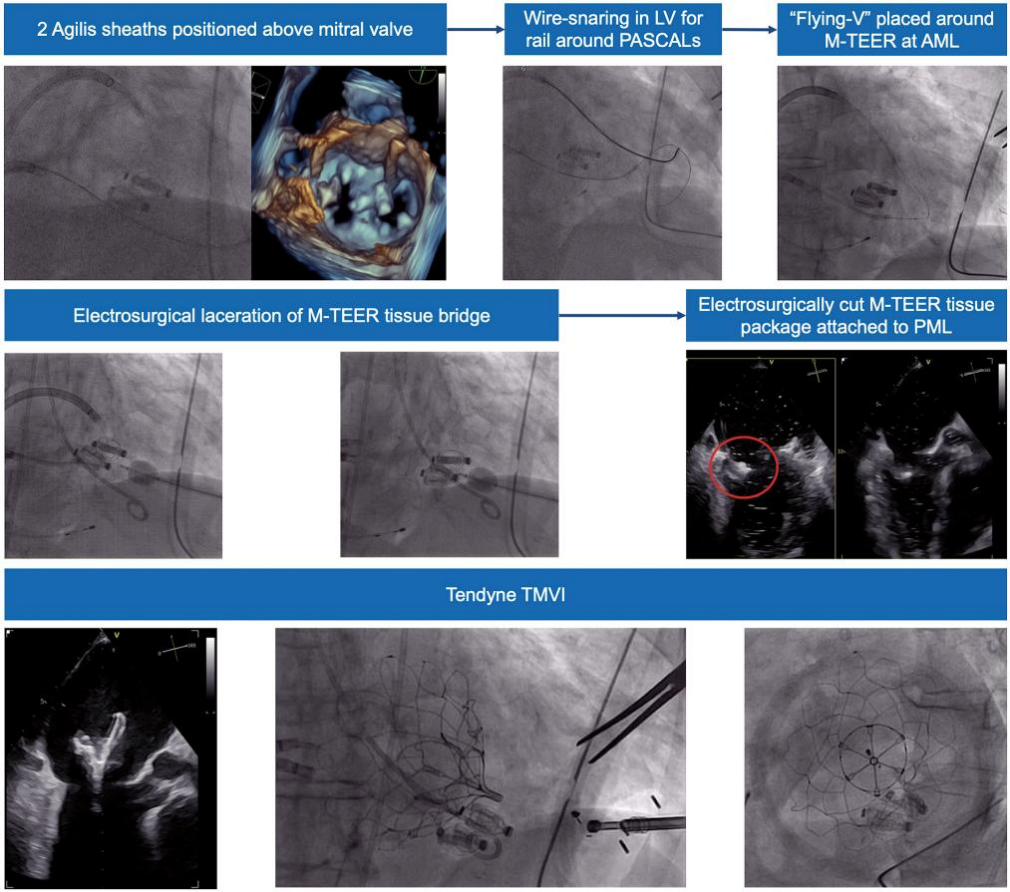
Procedure of electrosurgical laceration and stabilization of two PASCAL devices and subsequent transcatheter mitral valve implantation using Tendyne. AML, anterior mitral leaflet; LV, left ventricle; M-TEER, mitral transcatheter edge-to-edge repair; PML, posterior mitral leaflet; TMVI, transcatheter mitral valve implantation.

The procedure was performed under general anaesthesia and a transapical surgical approach was prepared. A 24 Fr DrySeal sheath (W.L. Gore & Associates, Newark, DE, USA) was placed in the femoral vein for dual guiding sheath insertion. Two 8.5 Fr steerable Agilis NxT Dual Reach guiding sheaths (Abbott Vascular, Abbott Park, IL, USA) were positioned transeptally. Heparin was administered until an ACT > 300 s was reached. The medial orifice of the double-orifice mitral valve was traversed by a Swan-Ganz ballon catheter through the first Agilis. The second sheath was then navigated towards the lateral orifice. Meanwhile, the soft, unexpanded portion of the 27-45 EN Snare system (Merit Medical, South Jordan, UT, USA), guided by a JR4 catheter, was employed to traverse the lateral ostium. Following the placement of an Astato 0.014 coronary guidewire (Asahi Intecc, Aichi, Japan) via the medial system, the Swan-Ganz catheter was replaced with a JR4 guide catheter. The Astato wire was directed towards the lateral snare and advanced, and once the basket was exposed, it was used to capture the Astato wire. Next, the guidewire was selectively stripped of its insulation and bent externally to form a ‘flying-V’ configuration. By pulling the captured Astato wire through the lateral Agilis/multipurpose system, the flying V was positioned directly next to the clips on the AML side. Using echocardiographic and fluoroscopic guidance, the flying V was placed directly adjacent to the tissue bridge of the two PASCAL devices. It is critical to encircle the AML of two PASCAL devices to ensure that, after the burning process, the two devices remain connected to the PML and can be pushed into a posterior pocket by the prosthesis. The PML and the attached PASCAL devices are retracted towards the ventricle by the subvalvular chordae and blood flow towards the left ventricle (LV). To ensure rapid procedure progress after the AML burn, the ventricular apex was punctured, an eight French sheath was successfully introduced and a Fogarthy catheter was placed in the LV to prepare for TMVI. Subsequently, a continuous 70 W current was applied to the flying V wire with gentle traction on the lateral and medial catheters. Following a successful laceration, with both PASCAL devices still attached to the PML, TMVI placing a Tendyne prosthesis intra-annularly was performed (*Figure 4* and Supplementary material online, *Video S2*).

Echocardiography showed excellent haemodynamics with no paravalvular leakage and a transvalvular mean gradient of 4.0 mmHg. At 30-day clinical follow up the patient reported a substantial relief of symptoms.

## Discussion

Mitral regurgitation is a common clinical entity and to date, the ESC Guidelines recommend surgical mitral valve repair whenever feasible.^4^ However, great advances regarding percutaneous M-TEER solutions have been made over the last decade and recent data show excellent safety and promising clinical outcomes through 5-years of follow up.^5^ Moreover, a retrospective analysis suggests that TEER for severe MR is associated with lower incidence of cardiovascular death, stroke, and pacemaker implantation compared with surgical mitral valve replacement.^6^

In light of these findings, it is expected that the number of M-TEER procedures will increase and consequently the incidence of recurrent MR requiring adequate treatment. Due to space constraints, elevated inflow gradients, and progressive calcification of the valve leaflets, further treatment with edge-to-edge-techniques is often prohibitive and innovative electrosurgical methods like ELASTA must be considered. Limited experience with ELASTA and the complex anatomy of the mitral valve along with previously implanted PASCAL devices present significant challenges for interventional cardiologists. CT analyses are essential for procedural planning and AI-based programs bear the potential to significantly enhance and refine these evaluations, as exemplified in the present case. Automatic and rapid imaging analyses may allow for a substantially more detailed assessment in a still timely manner. The additional parameters provided by the software may refine precision of procedures like ELASTA, a key aspect to standardize such procedures as a part of lifetime management for MR patients.

## Conclusions

To our knowledge, this case represents the first ELASTA of 2 PASCAL devices followed by Tendyne TMVI. Moreover, it represents the first clinical experience with an AI-based software that supports pre-procedural CT evaluation for such procedures. In conclusion, our report highlights the safety and feasibility of ELASTA in the context of complex mitral valve anatomy with two previously implanted PASCAL devices. This is particularly important as the increasing number of M-TEER procedures is expected to result in a significant rise in recurrent MR, for which a standardized approach has not yet been established. Refined and precise planning for such procedures, potentially simplified and standardized by implementation of automatic AI-based software, will be fundamental to establish treatment pathways for adequate lifetime management of MR patients.

## Supplementary Material

ytaf202_Supplementary_Data

## Lead author biography

Dr Nienaber graduated from high school in 2013. He completed medical studies at the Medical Faculty of the University of Buenos Aires in Argentina and the Medical Faculty of the University of Cologne in Germany in 2020. Since September 2020, he is employed at the Clinic III for Internal Medicine of the University Hospital Cologne.

## Notes

**Consent:** The patient gave his consent in accordance with the COPE guidelines for the material presented to appear in the publication above and related publications. It was explained to him that the material has educational and scientific value and that the publication may help to improve the care that others will receive in the future; however, the patient did and will not receive any financial benefit. He has had the opportunity to see and read the material submitted for publication, and it was explained that the final publication may differ in style, grammar, consistency, and length.

**Funding:** None declared.

## Date availability

The data underlying this article are available in the article and in its online Supplementary material.
